# Arbitration between controlled and impulsive choices

**DOI:** 10.1016/j.neuroimage.2014.12.071

**Published:** 2015-04-01

**Authors:** M. Economides, M. Guitart-Masip, Z. Kurth-Nelson, R.J. Dolan

**Affiliations:** a Wellcome Trust Centre for Neuroimaging, Institute of Neurology, University College London, London WC1N 3BG, UK; b Ageing Research Centre, Karolinska Institute, SE-11330 Stockholm, Sweden; c Max Planck Centre for Computational Psychiatry and Ageing Research, University College London, London, UK

**Keywords:** Decision-making, fMRI, Self-control, Value, Ventromedial prefrontal cortex, Striatum

## Abstract

The impulse to act for immediate reward often conflicts with more deliberate evaluations that support long-term benefit. The neural architecture that negotiates this conflict remains unclear. One account proposes a single neural circuit that evaluates both immediate and delayed outcomes, while another outlines separate impulsive and patient systems that compete for behavioral control. Here we designed a task in which a complex payout structure divorces the immediate value of acting from the overall long-term value, within the same outcome modality. Using model-based fMRI in humans, we demonstrate separate neural representations of immediate and long-term values, with the former tracked in the anterior caudate (AC) and the latter in the ventromedial prefrontal cortex (vmPFC). Crucially, when subjects' choices were compatible with long-run consequences, value signals in AC were down-weighted and those in vmPFC were enhanced, while the opposite occurred when choice was impulsive. Thus, our data implicate a trade-off in value representation between AC and vmPFC as underlying controlled versus impulsive choice.

## Highlights

• vmPFC evaluates action by considering immediate rewards and future consequences.
• Anterior caudate evaluates action only by considering immediate rewards.
• These value representations form dissociable systems that compete for control.
• Self-control is linked to fluctuations in value representation within each system.

## Introduction

Everyday occurrences often involve negotiating immediate temptations whose consumption might jeopardize long-term goals. A common instance is where the prospect of a large immediate reward is coupled with a harmful yet delayed consequence, such as enjoying a cigarette that can imperil long-term health. Behavioral findings suggest that in this context the desire for a hedonic payoff competes with the intent to act with foresight (Baumeister et al., 1998; Hare et al., 2009; Hofmann et al., 2009), demanding self-control.

A longstanding notion in psychology is that resisting temptation involves a competition between two competing systems (Hofmann et al., 2009; Hofmann and Van Dillen, 2012). In support of this idea, several experiments have found evidence for a trade-off between separate neural systems that preferentially activate when choice is driven by immediate and delayed rewards respectively (McClure et al., 2004; Tanaka et al., 2004). These systems are thought to guide choice by encoding value on opposing time-scales, though it is unclear whether their selective involvement reflects the tracking of other decision components.

An alternative perspective, particularly within neuroeconomics, suggests that choice is driven by a single system that represents both immediate and delayed decision outcomes. In dietary choice paradigms, where individuals choose between foods that vary along a scale of healthiness and tastiness (Hare et al., 2009, 2011), neuroimaging supports a role for the ventromedial prefrontal cortex (vmPFC) in integrating both components of value (Hare et al., 2009; Rangel, 2013). This is reinforced by other evidence that a common vmPFC–striatal circuit tracks the subjective value of choice options (Kable and Glimcher, 2007). The divergence between these two perspectives remains largely unresolved.

Here, we designed a novel paradigm that required subjects to accept or reject offers with known immediate value, presented sequentially within a trial. The probability of receiving large or small offers depended on past actions, such that an early acceptance of a large immediately available offer harmed long-term earnings by diminishing the opportunity for future rewards. Thus, maximizing long-run earnings sometimes required rejecting seemingly attractive offers associated with a high immediate payoff. In contrast to previous paradigms, long-run consequences were fully defined within a single outcome modality based on knowledge of the formal structure of the task. In this way our design permitted us to decorrelate immediate from long-term value across offers, where the latter includes the delayed consequences of acting. We used model-based functional magnetic resonance imaging (fMRI) to investigate the neural representation of each value component and linked this to a disposition for controlled versus impulsive action.

## Materials & methods

### Subjects

23 adults participated in the experiment (9 males and 14 females; age range 18–26; mean 21.2, SD = 2.33 years). All were healthy, reporting no history of neurological, psychiatric or other current medical problems. Subjects provided written informed consent to partake in the study, which was approved by the local ethics board (University College London, UK).

### Training paradigm

In a conditioning phase, performed outside of the scanner, subjects learnt stimulus–reward associations between a set of three differently colored rectangular cues and their respective reward values. Each colored rectangle corresponded to one of three possible outcomes involving receipt of 3, 5, or 7 tokens, randomized across individuals. Subjects were instructed that each token would translate into a fixed sum of money at the end of the experiment. Each trial began with a central fixation cross presented for 1000 ms, followed by a presentation of a random pair of colored cues, one appearing to the left one to the right of the screen. Subjects had a 2000 ms time-window to choose between these two boxes via a left or right button press, followed by presentation of the outcome of their choice for 1000 ms. The outcome was a written message indicating the total number of tokens won. Subjects were instructed to explore all options until they were confident that they had learnt all three associations, after which they should choose the box from the pair with the higher value. Each trial was defined as either correct if the subject chose the more valuable of the two options, and incorrect if the less valuable option was chosen. To ensure adequate learning, performance was calculated over six bins of twenty trials, with all subjects reaching a performance criterion of ≥ 90% by trial 60 onwards. Subjects were asked to verbally communicate the nature of the learnt associations.

### Task paradigm

On every trial subjects were presented with a random sequence of trained stimuli (see the Training paradigm section), appearing individually and sequentially, with a variable inter-stimulus interval (750–1250 ms). Each stimulus, presented for 1500 ms, constituted an offer requiring either a go response to win the relevant number of tokens or a nogo response which lead the player to forego monetary gain. However, a restriction was placed on the number of offers that could be exploited for reward on any given trial. Specifically, subjects were instructed that they could receive between 7–9 offers out of which between 4–6 could be accepted. The precise offer number and acceptance budget were drawn randomly and independently on every trial under a uniform distribution, and thus every combination was equally likely. A green circle on the top central portion of the screen turned red to indicate that a player had exhausted their go budget, after which nogo responses were enforced for any remaining offers.

Importantly, the value of each offer was probabilistic and governed by a set of explicitly instructed contingencies. At trial onset, each offer had an independent and equal probability of being worth 3, 5 or 7 tokens {0.33 0.33 0.33 (for 3, 5 and 7 respectively)}. Excluding the first offer, if a player accepted a value 7 offer before rejecting three or more previous offers the distribution would shift such that every future offer would have a probability distribution greatly in favor of value 3 {0.9 0.05 0.05}. Similarly, excluding the first offer, if a player accepted a value 5 offer before rejecting three or more previous offers the distribution would shift such that every future offer would have a probability distribution moderately in favor of value 3 {0.5 0.25 0.25}. The probability distribution was updated according to the choice made on the most recent offer. Thus a player had to consider both the immediate and long-term consequences of a go response in order to maximize payoff across a trial. Following the last offer, an outcome displaying the total number of offers won was presented on the screen for 2500 ms.

All subjects received 1 block (36 trials) of training outside the scanner in order to familiarize themselves with the task attributes and to diminish learning in the scanner. Subsequently, 108 trials were completed in the scanner across three sessions of 36 trials. The number of tokens won across sessions was summed and converted to a cash prize.

Due to the complex nature of the task, subjects were probed to ensure that they had currently understood the nature of the contingencies that linked actions to switches in the distribution of offers. Specifically we constructed a written set of eleven hypothetical trials, where for each trial subjects were asked to indicate their belief in the current offer distribution given a history of specific offers and actions. For example, “What is the probability of the next offer being worth 5 tokens given that a value 7 offer was accepted at the third index and no offers had previously been rejected?”. Subjects who failed to report the correct offer distribution for at least nine of the eleven trials were excluded from the experiment.

### Behavioral data analysis

#### Within-trial modulation of choice

Within a trial, a player transitions through a number of discrete states dependent on three fluctuating variables, the number of offers already seen, the number of accepts already expended and the current offer distribution. To assess how the probability of accepting a given offer fluctuated as a function of these variables, we split trials by offer index (i.e. 1–9), the number of offers already rejected (i.e. 0–8), and the current offer distribution, calculating the probability of accepting at every possible permutation (Fig. 2A). Note that we only display behavior corresponding to offers where the probability distribution is equal given that choice under this contingency is most relevant to the questions of interest. The probability of accepting at every state was averaged across all participants. For display purposes, we discard cells with less than a total of 15 data points.

#### Robust logistic regression

In order to confirm our hypothesis that both immediate and long-term values show independent effects on choice (a prediction that remains agnostic about the underlying neural architecture generating choice), we used a robust logistic regression to model the dependence of a go/nogo response (across all choice data) on immediate and long-term values in a model in which both regressors competed for variance. The algorithm implemented used iteratively reweighted least squares with a logistic weighting function. We performed one-sample t-tests on the resulting beta coefficients across subjects. A positive beta implies that subjects are more likely to go when value is high. We also repeated the logistic regression using choice data confined to offers that entered the imaging analysis (see Fig. 2A, middle panel, yellow boxes).

#### Computational modeling

A major interest here is the extent to which subjects utilize estimates of immediate and long-term values to guide choice. We utilized computational modeling to evaluate evidence that choice was guided purely by immediate value, purely by (the optimal) long-term value, or by a corresponding trade-off. Each model calculated the value of accepting an offer which was passed through a sigmoid function (σ) to determine action probabilities as follows:

$$P_{A}=\;\frac{1}{1+\exp\left(-\tau \cdot V_{A}\right)}$$

where *V_A_* is the expected value of accepting an offer, and *τ* is a temperature parameter that governs the stochasticity of choices.

##### Immediate reward model

We conjectured that subjects might choose on the basis of immediate value, disregarding the downstream consequences associated with prematurely accepting high (face) value offers, whereby

$$V_{A}=IR-c_{1}$$

where *IR* is the face value of the current offer and *c*_1_ represents a value intercept.

*IR*, *c*_1_ and *τ* (the temperature parameter of the associated sigmoid function) were fit by maximum likelihood estimation (see the Model fitting & comparison section).

##### Optimal model

We built a model that calculated the optimal decision at each offer, where optimal is defined as maximizing expectation of total reward delivery in the trial. The model assumes correct knowledge of the structure of the task. The current state of the task was defined by three belief distributions: **O**, over *o*, the number of offers remaining, **A**, over *a*, the number of accepts remaining, and **M**, the probability distribution governing the value of the forthcoming offer. The expected value of being in a state was:

$$\begin{array}{c} SV\left(O,A\right)=\sum_{r=\left\{3,5,7\right\}}M_{m}\left(r\right)\cdot \max\left\{P\left(o>1\right)\cdot SV\left(O^{\prime },A\right),\right)\\ \;\left(r+P\left(o>1\right)\cdot P\left(a>1\right)\cdot SV\left(O^{\prime },A^{\prime }\right)\right\} \end{array}$$

where ***O′*** is defined by

$$P\left(O^{\prime }=o\right)=\frac{P\left(O=o+1\right)}{\sum_{o}P\left(O=o+1\right)}$$

and ***A′*** is defined analogously. Thus going from ***O*** to ***O′*** or ***A*** to ***A′*** updates the probability distribution such that it remains uniform but shifts to the left. Note that calculating the recursive *SV* function was effectively a search through a tree of all possible moves. The recursion ends when *P*(*o* > 1) or *P*(*a* > 1) is 0, and *SV* is not evaluated.

**M** is defined by three discrete probability distributions as follows:

M_0_ = {0.33 0.33 0.33}
M_1_ = {0.50 0.25 0.25}
M_2_ = {0.90 0.05 0.05}

At trial onsets, *m* = 0, or and is updated according to the following rules:

If we are on the first offer, or 3 offers have previously been rejected, *m* doesn't change.
Otherwise, if a value 5 offer is accepted, *m* = 1, and if a value 7 offer is accepted, *m* = 2.

At each offer the model calculated the value of rejecting,

$$V_{R}=P\left(o>1\right)\cdot SV\left(O^{\prime },A\right)$$

and the future value of accepting,

$$V_{AF}=P\left(o>1\right)\cdot P\left(a>1\right)\cdot SV\left(O^{\prime },A^{\prime }\right)\text{.}$$

The expected value difference between accepting and rejecting, *EV*, was calculated as,

$$EV=V_{AF}+IR-V_{R}$$

where *IR* represents the (face) value of the current offer.

*EV* was passed through a sigmoid function to determine *P_A_*, the probability of a go response (see above).

##### Trade-off model

Based on evidence from our task and other studies utilizing similar paradigms (Economides et al., 2014; Hare et al., 2009, 2014; Kable and Glimcher, 2007), that immediate and long-term values exert independent influences on behavior, we hypothesized that subjects' choices might involve a trade-off between the two values. This hypothesis refers to subjects' behavior and remains agnostic as to the neural architecture that actualizes choice. In this regard, a choice pattern that utilizes both values could emerge either from a single integrated system or a dual-system architecture respectively. We specified a model in which immediate and long-term values both contributed independently to the calculation of expected value (TV, trade-off value), whereby the associated trade-off was captured by a single parameter that governed the weight placed on either value as follows:

$$TV=\left(EV\cdot c_{1}\right)+\;\left(IR-c_{2}\right)\;\cdot \left(1-\;c_{1}\right)$$

where *EV* is the expected, or long-term value, derived from the optimal model (see above), *IR* is the (face) value of the current offer, *c*_1_ governs the nature of the trade-off, and *c*_2_ represents a value intercept.

In addition, it seemed reasonable to assume that subjects might trade-off immediate and long-term values differently depending on the face value of the current offer. We therefore specified a second trade-off model in which a separate trade-off parameter governed the weight placed on immediate and long-term values for each face value (3, 5 and 7).

#### Model fitting & comparison

As described in previous reports (Guitart-Masip et al., 2012; Huys et al., 2011) we used a hierarchical Type II Bayesian (or random effects) procedure using maximum likelihood to fit simple parameterized distributions for higher level statistics of the parameters. Since the values of parameters for each subject are ‘hidden’, this employs the Expectation–Maximization (EM) procedure. Thus on each iteration the posterior distribution over the group for each parameter is used to specify the prior over the individual parameter fits on the next iteration. For each parameter we used a single distribution for all participants. Before inference, all parameters were suitably transformed to enforce constraints (log and inverse sigmoid transforms).

Models were compared using the integrated Bayesian Information Criterion (iBIC), where small iBIC values indicate a model that fits the data better after penalizing for the number of parameters. Comparing iBIC values is akin to a likelihood ratio test (Kass and Raftery, 1995).

### fMRI data acquisition

fMRI was performed on a 3-Tesla Siemens Quattro magnetic resonance scanner (Siemens, Erlangen, Germany) with echo planar imaging (EPI) and a 32-channel head coil. Functional data was acquired over three sessions containing 280 volumes with 48 slices (664 volumes total). Acquisition parameters were as follows: matrix = 64 × 74; oblique axial slices angled at − 30° in the antero-posterior axis; spatial resolution: 3 × 3 × 3 mm; TR = 3360 ms and TE = 30 ms. The first five volumes were subsequently discarded to allow for steady state magnetization. Field maps were acquired prior to the functional runs (matrix = 64 × 64; 64 slices; spatial resolution = 3 × 3 × 3 mm; gap = 1 mm; short TE = 10 ms; long TE = 12.46 ms; TR = 1020 ms) to correct for geometric distortions. In addition, for each participant an anatomical T1-weighted image (spatial resolution: 1 × 1 × 1 mm) was acquired for co-registration of the EPIs.

During scanning peripheral measurements of subject pulse and breathing were made together with scanner slice synchronization pulses using the Spike2 data acquisition system (Cambridge Electronic Design Limited, Cambridge, UK). The cardiac pulse signal was measured using an MRI compatible pulse oximeter (Model 8600 F0, Nonin Medical, Inc., Plymouth, MN) attached to the subject's finger. The respiratory signal (thoracic movement) was monitored using a pneumatic belt positioned around the abdomen close to the diaphragm.

### fMRI data analysis

Data were pre-processed and analyzed using SPM8 (Wellcome Trust Centre for Neuroimaging, UCL, London). Functional data were bias corrected for 32-channel head coil intensity inhomogeneities, realigned to the first volume, unwarpped using individual fieldmaps, co-registered to T1w images, spatially normalized to the Montreal Neurology Institute (MNI) space (using the segmentation algorithm on the T1w image with a final spatial resolution of 1 × 1 × 1 mm) and smoothed with an 8 mm FWHM Gaussian kernel. The fMRI time series data were high-pass filtered (cutoff = 128 s) and whitened using an AR(1)-model.

For each subject we computed a statistical model by applying a canonical hemodynamic response function (HRF) combined with time and dispersion derivatives. Using an in-house Matlab toolbox (Hutton et al., 2011) we constructed a physiological noise model to account for artifacts that take account of cardiac and respiratory phases as well as changes in respiratory volume. This resulted in a total of 14 regressors which were sampled at a reference slice in each image volume to give a set of values for each time point. The resulting regressors were included as confounds in our GLM at the first level (see below).

#### GLM 1

In order to investigate regions tracking immediate or long-term value, we designed a GLM that allowed us to explore the BOLD response to a subset of offers for which immediate and long-term values were most decorrelated, corresponding to offers between indexes 2 and 3 within a trial (see Fig. 2A, middle panel, yellow boxes). We split these offers contingent on their face value, such that each value (3, 5 and 7) was modeled as a separate regressor. Although these offers were selected from neighboring states (meaning that for any given (face) value, the long-term value of a go response was similar), each onset regressor was parametrically modulated by long-term value (from the optimal model) so as to account for variance associated with a difference in the current state. Additional regressors included the onsets of all within-budget offers outside of the yellow box in Fig. 2A, parametrically modulated by both immediate and long-term values, all out-of-budget offers (for which nogo responses were enforced), parametrically modulated by immediate value, the onset of go responses (button presses) across the entire experiment, so as to explain away motor-related activity, and the onset of trial outcomes (parametrically modulated by tokens won). Regressors of no interest included 6 movement-related covariates (the 3 rigid-body translations and 3 rotations resulting from realignment) and 14 physiological regressors (6 respiratory, 6 cardiac and 2 change in respiratory/heart rate). All regressors were modeled as stick functions with a duration of zero and convolved with a canonical form of the hemodynamic response function (HRF) combined with time and dispersion derivatives.

To explore the BOLD response to the onset of value 3, 5 and 7 offers when immediate and long-term values were decorrelated, we conducted a random-effects one-way ANOVA at the second level, with a single factor (face value) and 3 levels (3, 5, 7), containing individual subject first-level contrast images corresponding to the first three onset regressors from our GLM. We constructed functional ROIs (fROIs) from clusters that survived small volume correction for a prior volume of interest (see the Anatomical volume of interest section) using the MarsBar toolbox (v. 0.42) for SPM. We extracted mean parameter estimates from each fROI for our three onset regressors of interest and performed post-hoc paired t-tests to explore differences in BOLD response between offer values. For display purposes, onset parameter estimates were normalized (mean centered). In addition, we specified the contrast {0 − 1 1} corresponding to the onset of value 3, 5 and 7 offers to explore regions that covaried with the demand for control. The latter was performed as a whole-brain analysis.

In order to test whether the BOLD response to value 7 offers in our four ROIs was related to choice, we correlated mean parameter estimates (corresponding to the value 7 onset regressor) extracted from each ROI, with the trade-off parameter captured by our model fitting procedure. This resulted in four independent correlations.

#### GLM 2

In order to quantify the extent to which the BOLD response was modulated by immediate and long-term values, we built a second GLM where we concatenated the first three regressors from GLM 1 (onsets of 3, 5 and 7-token offers) into a single regressor, and added a parametric modulator for immediate value, which was forced to compete for variance (and was thus not orthogonalized) with an overall (long-term) value modulator. All other regressors remained identical to those specified in GLM 1.

We extracted mean parameter estimates from each ROI for the immediate and long-term value parametric modulators. For each ROI and each parametric modulator (immediate and long-term values), we conducted Grubb's test to probe for extreme values so as to remove subjects who were significant outliers at a threshold of p < 0.05 (8 tests in total); however this did not result in the exclusion of any subjects. Finally we performed one sample t-tests at the second level on the resultant betas across the group.

#### GLM 3

In order to look for difference in value coding during correct and incorrect responses, we split our regressor corresponding to the onset of value 7 offers from GLM 1 into correct (nogo) responses and incorrect (go) responses. All other regressors remained equivalent.

We conducted a random-effects one-way ANOVA at the second level, with a single factor (accuracy) and 2 levels (correct, incorrect), containing individual subject first-level contrast images corresponding to the go 7 and nogo 7 onset regressors. We extracted parameter estimates from each ROI and performed post-hoc paired t-tests to explore a main effect of response accuracy. We note that only 15 out of 23 subjects had enough variance in their ability to respond accurately across trials, and thus the above analysis was restricted to these individuals.

### Anatomical volume of interest

We constructed an anatomical volume of interest (VOI) that included individual valuation regions of a prior interest for the purposes of small volume correction, effectively reducing the number of voxel-wise comparisons. This consisted of the entire vmPFC, caudate nucleus, putamen and ventral striatum (nucleus accumbens) (see Fig. S3). The vmPFC and dorsal striatum were defined as anatomical ROIs from the MarsBar toolbox (v. 0.42) for SPM. For the ventral striatum we used a group-average ROI derived from a diffusion tensor imaging connectivity-based parcellation of the right nucleus accumbens in humans, taken from Baliki et al. (2013). This ROI consisted of both the core and shell subcomponents of nucleus accumbens. The right region was flipped along the x-dimension in the MarsBar toolbox to obtain bilateral accumbens.

We constructed an anatomical volume of interest (VOI) that included individual valuation regions of a prior interest for the purposes of small volume correction, effectively reducing the number of voxel-wise comparisons. This consisted of the entire vmPFC, caudate nucleus, putamen and ventral striatum (nucleus accumbens) (see Fig. S3). The vmPFC and dorsal striatum were defined as anatomical ROIs from the MarsBar toolbox (v. 0.42) for SPM. For the ventral striatum we used a group-average ROI derived from a diffusion tensor imaging connectivity-based parcellation of the right nucleus accumbens in humans, taken from Baliki et al. (2013). This ROI consisted of both the core and shell subcomponents of nucleus accumbens. The right region was flipped along the x-dimension in the MarsBar toolbox to obtain bilateral accumbens.

### Functional regions of interest

We defined functional regions of interest (fROIs) from clusters that survived small volume correction for a pre-defined VOI (see above) when testing for regions tracking IR or EV using GLM 1 (see GLM 1 above). For the anterior caudate, we excluded voxels that fell outside of an anatomical ROI for bilateral caudate from the MarsBar toolbox. These fROIs were used for all remaining fMRI analyses. All ROI analyses were performed using the MarsBar toolbox (v. 0.42) for SPM.

## Results

**Fig. 1:**
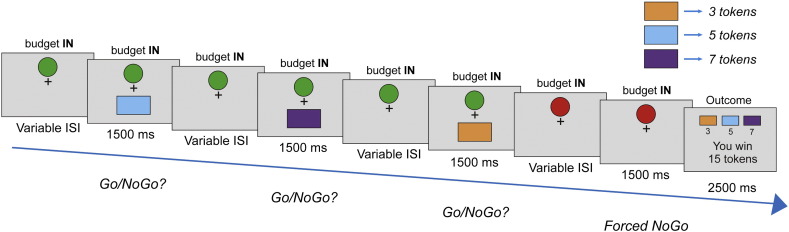
Task schematic. In the pre-scanning training (not shown), subjects learnt to associate three distinct color stimuli with a token value of 3, 5 or 7, with each token won translated into a cash prize at the end of the experiment. In the actual experiment proper (shown above), a player was presented with a sequence of stimuli, each constituting an individual offer. These offers required a go response to win or a nogo response to forego a gain. Crucially, a restriction was placed on the number of offers that could be exploited per trial sequence, such that on every trial a player could receive an overall amount of 7–9 offers but where only 4–6 (go budget) could be accepted, with every combination being equally likely. A green circle at the top central portion of the screen turned red to indicate players had exhausted their go budget, after which they passively observed the remaining sequence of outstanding offers. At trial onset, each offer had an equal probability of being the color associated with 3, 5 or 7 tokens {0.33 0.33 0.33, respectively}. With the exception of the first offer, if a player accepted a value 7 offer before rejecting at least three previous offers, the distribution would shift in favor of value 3 offers for the remainder of the sequence {0.9 0.05 0.05}. Likewise, if a player accepted a value 5 offer before rejecting at least three previous offers, the distribution would modestly shift in favor of value 3 offers {0.5 0.25 0.25}. The current distribution was updated based on the most recent action. Thus, an optimal player had to track the immediate reward environment as well as calculate overall (long-term) value by taking account of how an immediate go response might impact on future reward abundance, entailing often rejecting an offer associated with a large immediate reward.

In every trial, subjects received between 7–9 offers, but an imposition of a limited “budget” meant that they could only accept between 4–6 offers. Importantly, we penalized acceptance of the largest (7-token) and second largest (5-token) offers early in a trial by impoverishing remaining offers in that trial, where the penalty scaled with the face value of the current offer (see Fig. 1 and the Materials & methods section). Here, immediate value equates to the face value of each offer (3, 5 or 7 tokens), whereas long-term value represents the total expected utility from accepting. Thus, long-term value includes the face value, the cost of expending a unit of budget, and the cost of changing the future probability of reward. In some cases, total earnings could be maximized by rejecting 7-token but not 5-token offers. This is because the penalty associated with an accept response can be greater than the immediate payoff for 7-token, but not 5-token offers. In other words, the long-term value of a 7-token offer can sometimes be negative while nonetheless yielding the highest immediate payoff. Hence, immediate value was decorrelated from long-term value across offers, despite the former being a component of the latter.

Given the complexity behind the rules governing how actions shaped future offers, subjects were probed prior to scanning to ensure that they correctly understood the contingencies of the task (see the Materials & methods section). In brief, each subject was shown a series of hypothetical trials where they had to predict the probability of a forthcoming offer being a specific value, given a preceding sequence of offers and actions. All subjects demonstrated correct understanding of the task and were fully aware of the contingencies linking actions to states following careful instruction. In addition, in order to minimize effects of learning and uncertainty during scanning, subjects played one block (36 trials) of the task prior to performing the experiment in the scanner.

**Fig. S1:**
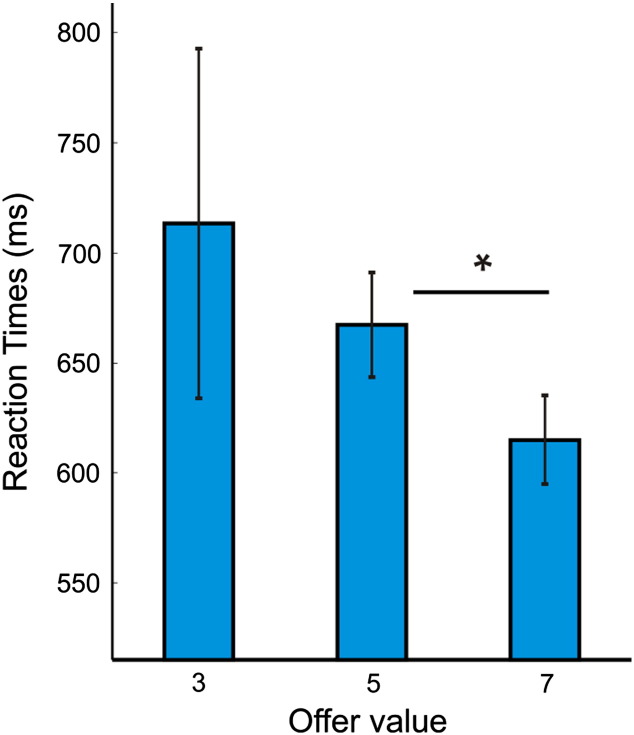
Reaction times for offers where the requirement for self-control is greatest. Here we plot average (mean) reaction times across the group (based on individual means), considering all ‘go’ responses for offers at the start of a trial where the requirement for self-control is greatest (see Fig. 2A, middle panel, yellow boxes). Note that these also represent the set of offers that entered our imaging analyses. Using Grubb's test for outliers, we excluded data from one subject whose mean reaction time for value 7 offers was significantly higher than the group (at a threshold of p < 0.05). Subjects were faster to accept token-value 7 offers compared to value 5 offers, suggestive of a prepotent attraction to 7-token cues. Vertical lines represent SEM. * indicates p < 0.05 (paired t-test).

**Fig. 2:**
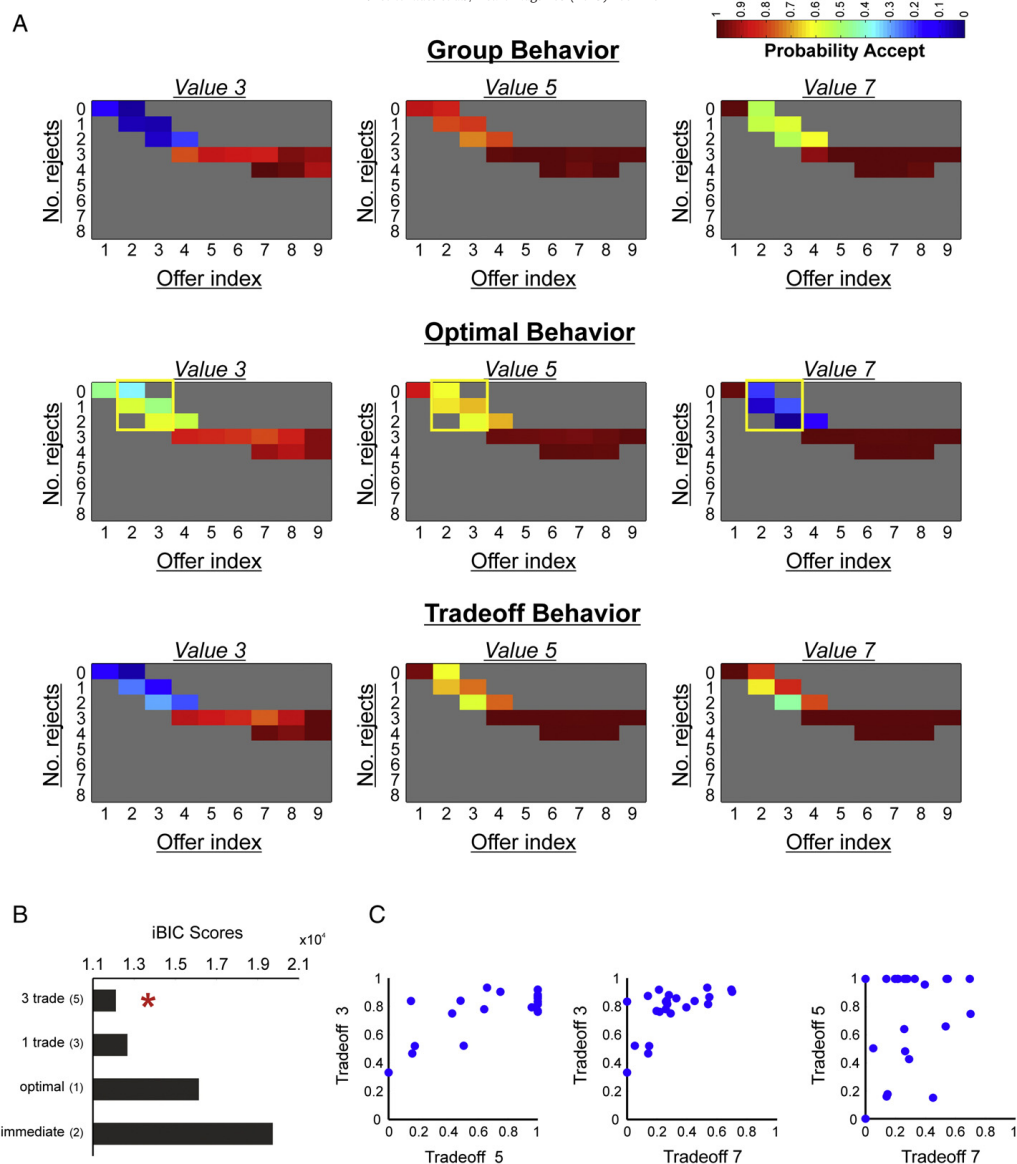
Behavioral results. (A) Plotted above are subjects' mean probability of offer acceptance as a function of the number of offers already seen (ranging from 1–9) and number of offers already rejected (ranging from 0–8) in a trial, split by offer value (3, 5, 7) (top panel). The spectrum runs from blue (p = 0) to red (p = 1). We stress that we have only plotted behavior corresponding to periods in a trial where the offer distribution is uniform and thus any penalty for prematurely accepting a high value offer has not yet been instantiated. Compared to an optimal model in which choice is dictated by correctly inferring long-term value (middle panel), subjects under-accept value 3 offers and over-accept value 7 offers at the start of trials (top panel; based on group mean data, n = 23). This discrepancy is rectified by a model in which immediate and long-term values trade off for behavioral control (lower panel). We note that the lower panel illustrates choice predicted by the trade-off model based on mean group parameter fits (n = 23). The yellow boxes in the middle panel demonstrate offers for which immediate and long-term values are maximally decoupled, and those for which all fMRI analyses are centered on. We note that for display purposes, we discarded cells with less than a total of 15 data points across subjects. (B) Model comparison showed that a model in which each offer value (3, 5, 7) is assigned a separate parameter that governs how much weight is placed on immediate versus long-term value in the associated trade-off fits behavior better than alternatives, indicated by its lowest iBIC score (3 trade). These alternatives included a model in which a single parameter governs the trade-off (1 trade), a model dependent on optimally inferring long-term value (optimal), and a model driven purely by immediate value (immediate). The number of free parameters is indicated in brackets for each model. (C) Pair-wise scatter plots show individually fit trade-off parameters (*c*_1_, see the Materials & methods section) from the winning model for 3 versus 5-token offers, 3 versus 7-token offers, and 5 versus 7-token offers. A trade-off value closer to 0 indicates that behavior is predominantly driven by immediate value, while a value closer to 1 indicates that behavior is predominantly driven by long-term value. Each circle represents one participant.

In Fig. 2A, we plot subjects' propensity to accept a given offer value (3, 5 or 7 tokens) as a function of the number of offers already received and the number already accepted/rejected in a trial. Note that here we only plot choice behavior for periods in a trial where the offer distribution is uniform and thus any penalty for prematurely accepting a high value offer has not been instantiated. Although self-control is multi-faceted, one important aspect is an ability to override one's impulses or prepotent responses (Gailliot and Baumeister, 2007). In our task, the requirement for this form of self-control is greatest near the start of a trial, where accepting a large immediate offer has detrimental future consequences (see Fig. 2A, middle panel, yellow boxes). In this part of a trial, we found that subjects under-chose 3-token offers and over-chose 5 and 7-token offers, as compared to an optimal model (Fig. 2A). Moreover, considering the same set of offers, subjects were faster to accept 7-token offers compared to 5-token (paired t(21) = 2.66, p = 0.015) offers when they chose to enact a ‘go’ response, suggestive of a greater prepotent tendency to reap large immediate rewards (see Fig. S1, in which we plot the group-mean of average (mean) response times at the single subject level). We note that the large error bar for 3-token offers in Fig. S1 represents the fact that subjects rarely accepted 3-token offers in this part of a trial (with an average of just 2.7 data points across subjects compared to 33.8 and 25.7 for 5 and 7-token offers respectively).

In Fig. 2A, we plot subjects' propensity to accept a given offer value (3, 5 or 7 tokens) as a function of the number of offers already received and the number already accepted/rejected in a trial. Note that here we only plot choice behavior for periods in a trial where the offer distribution is uniform and thus any penalty for prematurely accepting a high value offer has not been instantiated. Although self-control is multi-faceted, one important aspect is an ability to override one's impulses or prepotent responses (Gailliot and Baumeister, 2007). In our task, the requirement for this form of self-control is greatest near the start of a trial, where accepting a large immediate offer has detrimental future consequences (see Fig. 2A, middle panel, yellow boxes). In this part of a trial, we found that subjects under-chose 3-token offers and over-chose 5 and 7-token offers, as compared to an optimal model (Fig. 2A). Moreover, considering the same set of offers, subjects were faster to accept 7-token offers compared to 5-token (paired t(21) = 2.66, p = 0.015) offers when they chose to enact a ‘go’ response, suggestive of a greater prepotent tendency to reap large immediate rewards (see Fig. S1, in which we plot the group-mean of average (mean) response times at the single subject level). We note that the large error bar for 3-token offers in Fig. S1 represents the fact that subjects rarely accepted 3-token offers in this part of a trial (with an average of just 2.7 data points across subjects compared to 33.8 and 25.7 for 5 and 7-token offers respectively).

Inline Supplementary Figure S1

Inline Supplementary Fig. S1 can be found online at http://dx.doi.org/10.1016/j.neuroimage.2014.12.071.

This pattern of choice is consistent with subjects being mindful of the future consequences of their actions, but nevertheless being over-susceptible to an influence of a current offer's face value. We therefore predicted that both immediate and long-term values (see the Materials & methods section for an explanation of how these are calculated) would independently influence behavior. Using logistic regression we indeed found that immediate (one-sample t(22) = 3.74, p = 0.001, mean b = 0.047) and long-term values (one-sample t(22) = 16.61, p < 0.001, mean b = 0.113) were significant predictors of choice, implying that behavior was neither exclusively optimal nor impulsive, but incorporated features of both traits.

Given evidence that immediate and long-term values exert a differential impact on action selection, we conjectured that a model encompassing a trade-off between each valuation would capture choice behavior. We used Bayesian model comparison to evaluate whether group behavior was driven exclusively by immediate value, by long-term value, or by a trade-off between the two (see the Materials & methods section). We found that a model in which each offer value (3, 5, 7) was assigned an independent trade-off parameter captured group-level choice best (Fig. 2B) (see Table S1 for a summary of best-fitting parameter estimates). Further, in order to provide an intuitive understanding of the goodness-of-fit of the different models tested, we calculated pseudo-r^2^ statistics for each model (see Table S2 for a summary), using Cox and Snell's method (Cox and Snell, 1989). We note that compared to a baseline intercept model, we identified a mean r^2^ value of 0.490 (95% CI [0.416, 0.563]) for the winning five-parameter model.

Given evidence that immediate and long-term values exert a differential impact on action selection, we conjectured that a model encompassing a trade-off between each valuation would capture choice behavior. We used Bayesian model comparison to evaluate whether group behavior was driven exclusively by immediate value, by long-term value, or by a trade-off between the two (see the Materials & methods section). We found that a model in which each offer value (3, 5, 7) was assigned an independent trade-off parameter captured group-level choice best (Fig. 2B) (see Table S1 for a summary of best-fitting parameter estimates). Further, in order to provide an intuitive understanding of the goodness-of-fit of the different models tested, we calculated pseudo-r^2^ statistics for each model (see Table S2 for a summary), using Cox and Snell's method (Cox and Snell, 1989). We note that compared to a baseline intercept model, we identified a mean r^2^ value of 0.490 (95% CI [0.416, 0.563]) for the winning five-parameter model.

Inline Supplementary Table S1

**Table S1:** Best-fitting group-level parameter estimates from the winning model, shown as median and quartiles across subjects. *τ* = inverse temperature, *c*_1_ = value intercept, *t*_3_ = trade-off for value 3, *t*_5_ = trade-off for value 5, *t*_7_ = trade-off for value 7.

	*τ*	*c*_1_	*t*_3_	*t*_5_	*t*_7_
25th percentile	1.856	5.160	0.763	0.488	0.160
Median	2.833	5.891	0.834	1.000	0.264
75th percentile	3.682	6.442	0.872	1.000	0.437

Inline Supplementary Table S1

Inline Supplementary Table S2

**Table S2:** Measure of goodness-of-fit, assessed via pseudo r^2^ (Cox and Snell, 1989), for the different models tested (shown as means with 95% confidence intervals).

r^2^	Immediate	Optimal	1 trade	3 trade
95% CI: lower	− 0.144	0.162	0.389	**0.416**
Mean	− 0.046	0.234	0.459	**0.490**
95% CI: upper	0.052	0.306	0.529	**0.563**

Inline Supplementary Table S2

Inline Supplementary Table S1 and Table S2 can be found online at http://dx.doi.org/10.1016/j.neuroimage.2014.12.071.

**Fig. S2:**
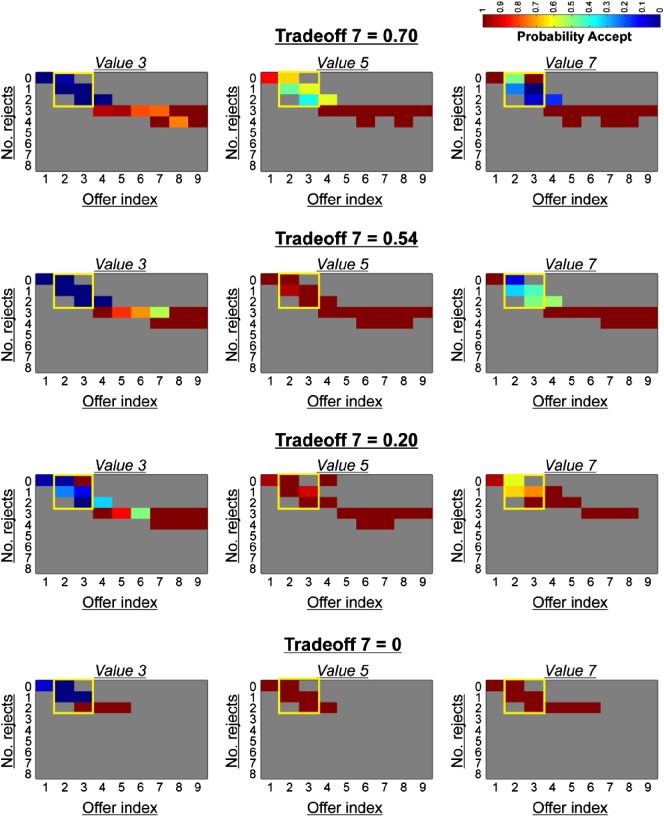
Exemplar single-subject choice demonstrating variance in the weighting placed on immediate versus long-term value. Plotted above are data from four individual subjects, where each row corresponds to one subject, showing the mean probability of offer acceptance as a function of the number of offers already seen (ranging from 1 to 9) and number of offers already rejected (ranging from 0 to 8) in a trial, split by offer value (3, 5, 7). The spectrum runs from blue (p = 0) to red (p = 1). The top row is representative of a subject who exercises greater self-control with respect to value 7 offers (trade-off 7 closer to 1), with the level of self-control decreasing in row-wise descending order (trade-off 7 equal to 0 in the bottom row). Those with a trade-off parameter closer to 1 are more likely to reject a value 7 offer when the requirement for self-control is greatest (see yellow boxes), whereas those with a trade-off parameter closer to 0 are more likely to accept a value 7 offer regardless of the associated consequences.

Hence, while subjects were considerate of future consequence, immediate rewards were (on average) overweighted across the experiment. The finding that subjects weight immediate and long-term values differently depending on face value is intuitive, as long-term value deviates from immediate value to a greater degree for some offers compared to others, and is thus sometimes harder to track. Indeed, the best-fitting trade-off parameters, which provide a measure of how strongly each player weighted immediate relative to long-term value for the three offers, strongly endorse this account (Fig. 2C). We provide examples of single subject data that illustrates how individuals varied widely in their ability to prioritize long-term value (in the face of large immediate rewards), ranging from the most controlled with respect to value 7 offers (trade-off parameter for value 7 closer to 1) to the most impulsive (trade-off parameter for value 7 closer to 0) (see Fig. S2).

Hence, while subjects were considerate of future consequence, immediate rewards were (on average) overweighted across the experiment. The finding that subjects weight immediate and long-term values differently depending on face value is intuitive, as long-term value deviates from immediate value to a greater degree for some offers compared to others, and is thus sometimes harder to track. Indeed, the best-fitting trade-off parameters, which provide a measure of how strongly each player weighted immediate relative to long-term value for the three offers, strongly endorse this account (Fig. 2C). We provide examples of single subject data that illustrates how individuals varied widely in their ability to prioritize long-term value (in the face of large immediate rewards), ranging from the most controlled with respect to value 7 offers (trade-off parameter for value 7 closer to 1) to the most impulsive (trade-off parameter for value 7 closer to 0) (see Fig. S2).

Inline Supplementary Figure S2

Inline Supplementary Fig. S2 can be found online at http://dx.doi.org/10.1016/j.neuroimage.2014.12.071.

Since immediate and long-term values exert distinct influences on choice we conjectured that these quantities would have dissociable representations in value sensitive brain regions. To test this, we used fMRI and implemented a GLM (see the Materials & methods section, GLM 1) in which each offer value (3, 5, 7) was modeled separately, but focused on a subset of offers where immediate and long-term values were maximally dissociable within any given trial (see Fig. 2A, middle panel, yellow boxes). In this set of offers, optimal behavior mandated strongly rejecting 7-token offers, strongly accepting 5-token offers, and weakly accepting 3-token offers. Thus, regions representing long-term (overall) value should display a BOLD signal profile that is attenuated for 7-token offers, boosted for 5-token offers and modestly boosted for 3-token offers. In contrast, regions that track immediate rewards should show a BOLD signal profile that increases linearly as a function of face value. In order to ensure that behavior in this subset of offers was similarly driven by both immediate and long-term (optimal) values, we repeated the aforementioned logistic regression but only included choices from within the yellow boxes in Fig. 2A (those which entered the imaging analysis). We again observed that both immediate (one-sample t(22) = 11.87, p < 0.001, mean b = 0.47) and long-term value (one-sample t(22) = 9.64, p < 0.001, mean b = 0.49) were significantly predictive of choice. Importantly, we modeled go responses as an independent regressor in all GLMs, and this spanned button presses across the entire experiment, including those corresponding to offers outside of the yellow box in Fig. 2A. Thus, any variance in activity attributed to cue onsets is independent from the generation of a motor response per se.

**Fig. S3:**
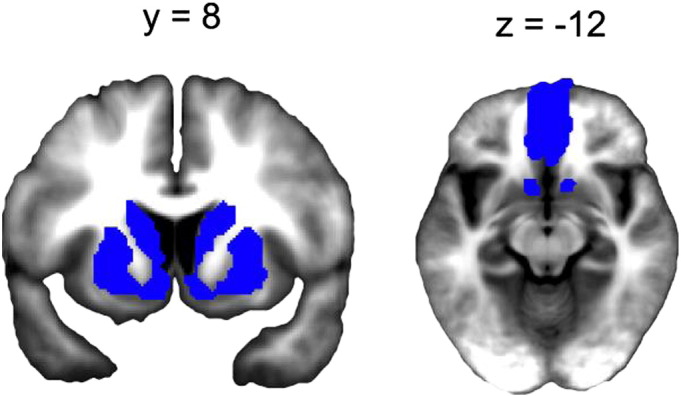
VOI. Volume of interest consisting of valuation regions of a priori interest used for small volume correction. Regions include vmPFC, bilateral caudate, bilateral putamen and bilateral ventral striatum (accumbens).

Given our a priori interest in responses within valuation regions, we generated a volume of interest (VOI; see Fig. S3) that included the ventromedial prefrontal cortex (vmPFC) (Balleine and Dickinson, 1998; Hare et al., 2009; Wunderlich et al., 2012), ventral striatum (Baliki et al., 2013; Guitart-Masip et al., 2012), caudate nucleus (Tricomi et al., 2004) and putamen (Brovelli et al., 2011) to constrain the search space and reduce the number of statistical comparisons. We used anatomical ROIs from the MarsBar toolbox (v. 0.42) for SPM and from previous research (see the Materials & methods section).

Given our a priori interest in responses within valuation regions, we generated a volume of interest (VOI; see Fig. S3) that included the ventromedial prefrontal cortex (vmPFC) (Balleine and Dickinson, 1998; Hare et al., 2009; Wunderlich et al., 2012), ventral striatum (Baliki et al., 2013; Guitart-Masip et al., 2012), caudate nucleus (Tricomi et al., 2004) and putamen (Brovelli et al., 2011) to constrain the search space and reduce the number of statistical comparisons. We used anatomical ROIs from the MarsBar toolbox (v. 0.42) for SPM and from previous research (see the Materials & methods section).

Inline Supplementary Figure S3

Inline Supplementary Fig. S3 can be found online at http://dx.doi.org/10.1016/j.neuroimage.2014.12.071.

**Fig. 3:**
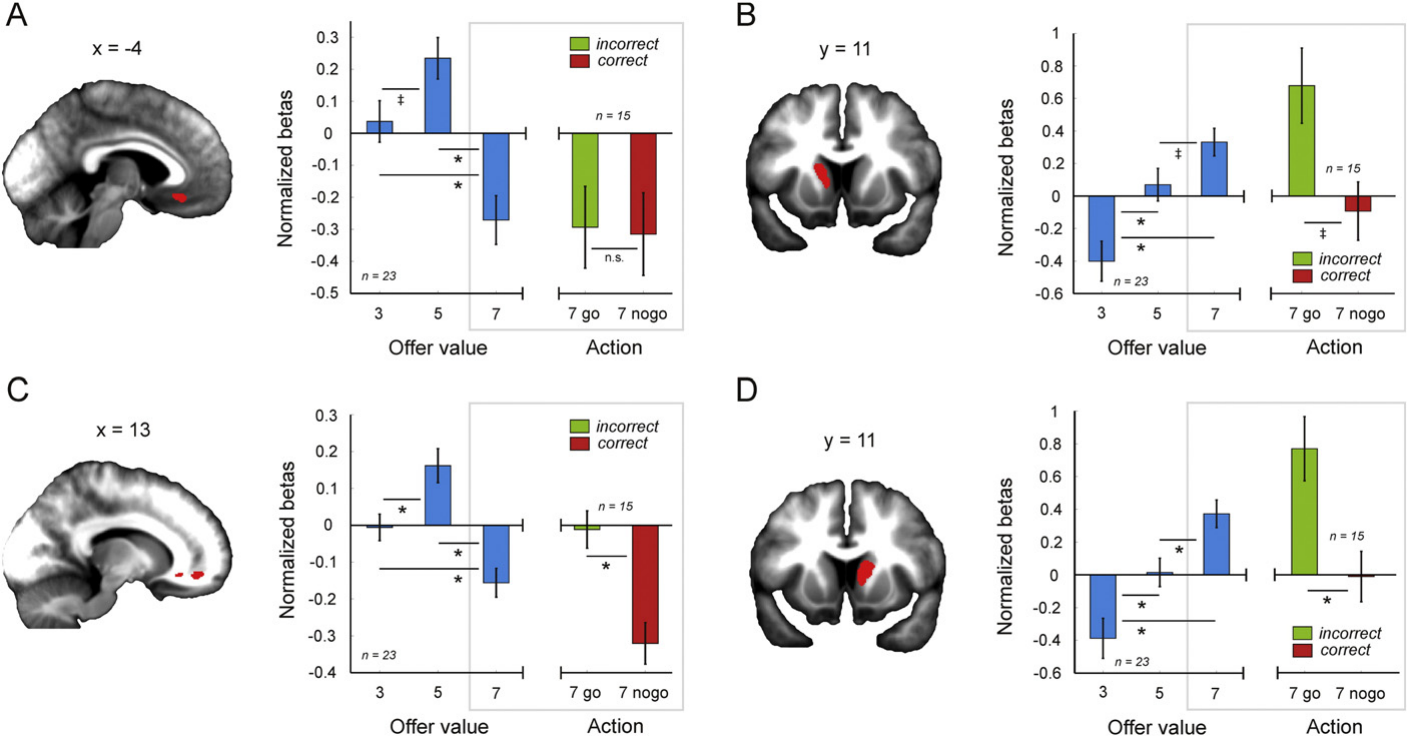
fMRI results. Clusters in ventral vmPFC (A) and lateral vmPFC showed greater activation in response to 5-token compared to 3-token offers, but a deactivation in response to 7-token relative to 3 and 5-token offers, consistent with 7-token offers having a negative overall (long-term) value (see yellow boxes, Fig. 2, panel A). By contrast, both left (B) and right (D) anterior caudate exhibited a linearly increasing response profile to the presentation of 3, 5 and 7-token offers, consistent with this region showing preferential sensitivity to immediate value. Furthermore, in trials where 7-token offers were impulsively accepted (7 go) compared to rejected (7 nogo), the representation of long-term value (for 7-token offers) was attenuated in lateral vmPFC (C) (less negative beta), while the representation of immediate value (for 7-token offers) was boosted in left (B) and right (D) anterior caudate (more positive beta). Vertical lines represent SEM. * indicates p = < 0.05; ^‡^ indicates p = 0.07; n.s. indicates not significant (paired t-tests).

When testing for regions that track long-term value (a contrast of {0 1 − 1} for 3, 5 and 7-token offers) we identified two clusters that survived small volume correction (SVC) for our VOI in vmPFC, including a ventral (Fig. 3A) and a more lateral portion (Fig. 3C). By contrast, when testing for regions that track immediate value (a contrast of {− 1 0 1} for 3, 5 and 7-token offers) we identified activation in both left (Fig. 3B) and right (Fig. 3D) anterior caudate nucleus that likewise survived SVC for our VOI. These clusters were then used to define functional regions of interest (fROIs) in vmPFC and anterior caudate for further analysis, which correspond to the regions displayed in Fig. 3.

To quantify the extent to which each fROI was preferentially driven by immediate versus long-term value, we constructed a second GLM that allowed us to regress both values against the BOLD signal within the same model, by collapsing offers into a single regressor and using immediate and long-term values as parametric modulators. Note that these regressors, for which the average correlation was r^2^ = 0.24, were not orthogonalized in our GLM and were forced to compete for variance (see the Materials & methods section, GLM 2). This analysis again showed that BOLD response in both the ventral (one sample t(22) = 3.21, p = 0.004, mean b = 0.148) and lateral (one sample t(22) = 4.07, p < 0.001, mean b = 0.100) vmPFC fROIs were driven by long-term value and not explained by immediate value (ventral: one-sample t(22) = − 0.913, p = 0.371, mean b = − 0.032); lateral: one-sample t(22) = − 0.263, p = 0.795, mean b = − 0.004). By contrast, BOLD in anterior caudate was driven predominantly by immediate value (left caudate: one-sample t(22) = 4.239, p < 0.001, mean b = 0.300; right caudate: one-sample t(22) = 4.504, p < 0.001, mean b = 0.227), though long-term value also contributed to signal variance (left caudate: one-sample t(22) = 2.21, p = 0.038, mean b = 0.127; right caudate: one-sample t(22) = 2.42, p = 0.024, mean b = 0.115) suggesting that it represented mixed value components.

It has been proposed that self-control involves a conflict between competing value systems (Hofmann et al., 2009; McClure et al., 2004; Tanaka et al., 2004), and this idea gains support from evidence that the brain draws on multiple systems when making decisions (Balleine, 2005; Daw et al., 2005; Dolan and Dayan, 2013). However, an alternative suggestion is that choice is governed by a common value system embedded in vmPFC (Hare et al., 2009) or a vmPFC–striatal network (Kable and Glimcher, 2007). Our finding that distinct representations of immediate and long-term values are tracked in the brain fits better with the idea of two competing value systems. However, we note that long-term value in our task includes both immediate and delayed components of value. Thus, our data is consistent with the notion that both value components are integrated within vmPFC (Economides et al., 2014; Hare et al., 2009). Importantly, if the separate encoding of immediate and long-term values is linked to the observed trade-off between these values during choice, we would expect between-subject variability in self-control to correlate with the strength with which long-term value was represented relative to immediate value. Specifically, a stronger representation of long-term relative to immediate value should track greater self-control. Indeed we would also expect that representations of immediate and long-term values would be altered in trials where subjects (incorrectly) accepted a 7-token offer compared to when subjects (correctly) resisted the temptation.

**Fig. 4:**
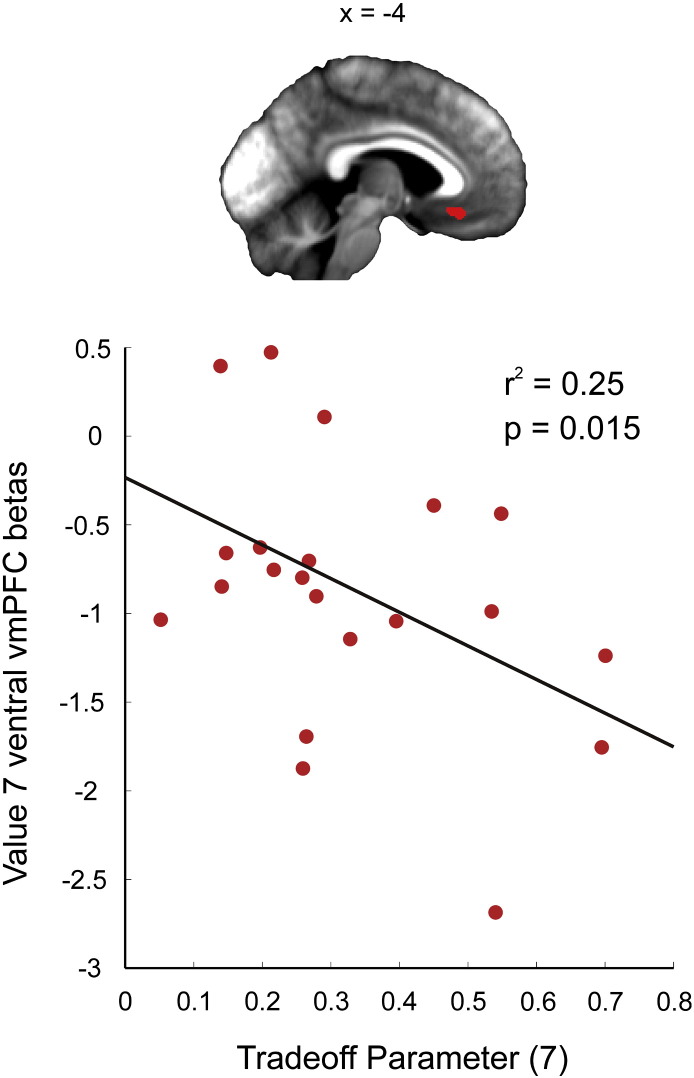
BOLD response in vmPFC correlates with a measure of self-control. When confronted with an offer associated with a high immediate value but low long-term value, between-subject variability in ventral vmPFC BOLD response to 7-token offers was tightly coupled with choice (r^2^ = 0.25, p = 0.015). The higher the signal in vmPFC, the more choice was driven by immediate value (more positive beta, trade-off parameter closer to 0). In contrast, the lower the signal in vmPFC, the more choice was driven by long-term value (more negative beta, trade-off parameter closer to 1).

To test the first prediction, we correlated parameter estimates for the onset of 7-token offers with the trade-off parameter which captures the weighting placed on immediate versus long-term value (for 7-token offers), for each of the four fROIs. The parameter estimates were derived from GLM 1 (see Fig. 3) and correspond to offers early in a trial where accepting a 7-token offer is detrimental overall despite yielding a large immediate reward. The weighting parameter effectively provides a measure of self-control for each individual player, although our task cannot distinguish whether subjects that over-accept 7-token offers do so because they overweight immediate value, or alternatively because they underweight the future consequences of accepting a high value offer (and thus miscalculate long-term value). Although we failed to identify any correlations that survived the most conservative correction for multiple comparisons (across the four fROIs), we nonetheless found a correlation between BOLD activation in response to 7-token offers and the trade-off parameter for 7-token offers in ventral vmPFC (r^2^ = 0.25, p = 0.015), that we report here as an exploratory result (see Fig. 4). In this region of vmPFC, a higher BOLD activation (implying a greater weighting on face value) in response to 7-token offers was linked to impulsively accepting (trade-off parameter fit closer to 0), while a lower BOLD activation (implying a greater weighting on long-term value) was linked to foregoing the option (trade-off parameter fit closer to 1).

In addition to observing variability in self-control between subjects, players were also highly variable in their ability to exercise control across trials. To test the prediction that trial-by-trial switches between controlled and impulsive choices are linked to a change in the representation of immediate or long-term value, we constructed a new GLM (see the Materials & methods section, GLM 3) where we split 7-token offers contingent upon whether they were (incorrectly) accepted or (correctly) resisted. This analysis once again focused on the subset of offers that fall inside the yellow box in Fig. 2A. We note that although a difference in BOLD between go and nogo at the time of cue onset could reflect a modulation of value representation, it could also be driven by the execution of a motor response in one condition and not the other. To control for this motor confound, we regressed out button presses using a motor regressor that included a large proportion of button presses from outside of the yellow box in Fig. 2A. However, we cannot fully exclude the possibility that any difference observed might be driven by the anticipation of an upcoming action.

Bearing in mind this caveat, we found that a BOLD response to a 7-token offer was on average less negative in lateral (paired t(14) = 3.734, p = 0.002, mean b difference = 0.309) but not ventral (paired t(14) = 0.103, p = 0.919, mean b difference = 0.0215) vmPFC, and more positive in bilateral anterior caudate (left caudate: paired t(14) = 2.068, p = 0.06, mean b difference = 0.772; right caudate: paired t(14) = 2.413, p = 0.030, mean b difference = 0.780) when subjects chose to incorrectly accept compared to correctly reject (Fig. 3). Thus, impulsive responses were accompanied by a weaker representation of long-term value within lateral vmPFC and an enhanced representation of immediate value in bilateral caudate, while optimal choices followed the reverse pattern. This profile implies that the representational fidelity of one aspect of a value computation may be promoted at the expense of the other.

Previous studies show that self-control recruits the dorsal prefrontal cortex (dPFC) with evidence suggesting that this region acts to initiate inhibitory control (Aron et al., 2004) or to modulate the representation of value within valuation regions (Diekhof and Gruber, 2010; Hare et al., 2009). While our primary interest with imaging was to identify the neural representations of immediate and long-term values, we conjectured that activity in dPFC might scale with the demand for control, and that this in turn may contribute towards the observed representations of value. Within a subset of offers at the start of each trial (yellow box in Fig. 2A), 7-token offers require amplified self-control relative to 3 and 5-token offers, as the immediate value of accepting a 7-token offer here is most decorrelated from the overall long-term value. Thus, the BOLD response in regions enacting ‘control’ should be enhanced in response to 7-token offers, diminished in response to 5-token offers, and modestly enhanced in response to 3-token offers. Note that this is the opposite profile to that observed in vmPFC that encodes long-term (overall) value (see Figs. 3A and C). We tested for this in a contrast ({0 − 1 1} for 3, 5 and 7-token offers) using GLM 1 where we identified activation in a frontal network including anterior cingulate cortex and right inferior frontal gyrus that survived whole-brain correction (see Table S3 for all areas).

Previous studies show that self-control recruits the dorsal prefrontal cortex (dPFC) with evidence suggesting that this region acts to initiate inhibitory control (Aron et al., 2004) or to modulate the representation of value within valuation regions (Diekhof and Gruber, 2010; Hare et al., 2009). While our primary interest with imaging was to identify the neural representations of immediate and long-term values, we conjectured that activity in dPFC might scale with the demand for control, and that this in turn may contribute towards the observed representations of value. Within a subset of offers at the start of each trial (yellow box in Fig. 2A), 7-token offers require amplified self-control relative to 3 and 5-token offers, as the immediate value of accepting a 7-token offer here is most decorrelated from the overall long-term value. Thus, the BOLD response in regions enacting ‘control’ should be enhanced in response to 7-token offers, diminished in response to 5-token offers, and modestly enhanced in response to 3-token offers. Note that this is the opposite profile to that observed in vmPFC that encodes long-term (overall) value (see Figs. 3A and C). We tested for this in a contrast ({0 − 1 1} for 3, 5 and 7-token offers) using GLM 1 where we identified activation in a frontal network including anterior cingulate cortex and right inferior frontal gyrus that survived whole-brain correction (see Table S3 for all areas).

Inline Supplementary Table S3

**Table S3:** Regions that track the demand for action control. Regions where BOLD covaried with the demand for control ({ 0 − 1 1 } for offers of token-values 3, 5 and 7 respectively) from GLM 1.

Name of region	Cluster FWE p value	MNI coordinates			Statistics	
		x	y	z	t value	Z score
Anterior cingulate	< 0.001	10	34	30	5.33	4.77
R supplementary motor area		8	16	66	3.69	3.42
L insula	0.013	− 33	15	0	5.09	4.49
R Insula	< 0.001	32	20	2	4.91	4.36
R inferior frontal gyrus		54	12	19	3.78	3.50
R parietal	< 0.001	58	− 43	34	4.23	3.85

Inline Supplementary Table S3

Inline Supplementary Table S3 can be found online at http://dx.doi.org/10.1016/j.neuroimage.2014.12.071.

**Fig. 5:**
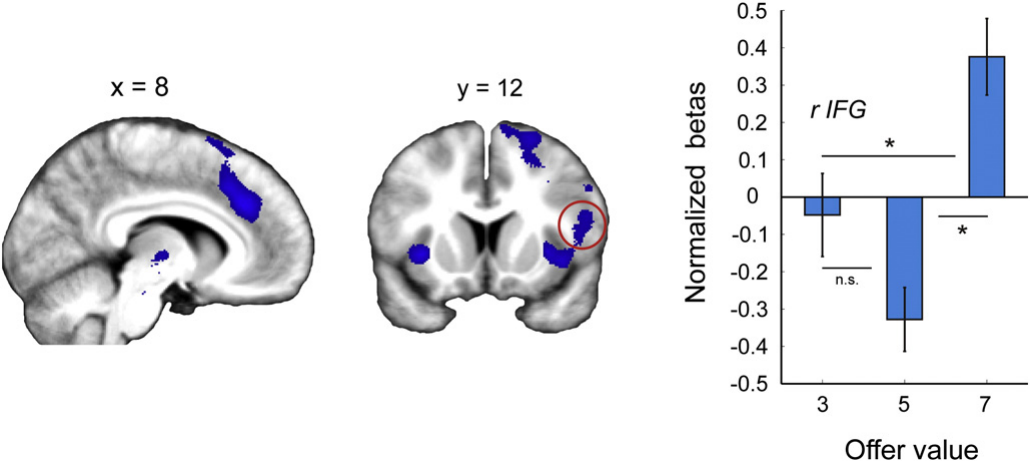
Activity in the brain scales with the requirement for action control. BOLD response within a frontal network including ACC, rIFG and bilateral insula cortex, was enhanced for 7-token offers compared to 3 and 5-token offers, and thus scaled with the demand for control. The betas for a region in the rIFG (circled in red) are plotted for illustration. Vertical lines represent SEM. * indicates p = < 0.05; n.s. indicates not significant (paired t-tests); see also Inline Supplementary Table S3. Activity in the brain scales with the requirement for action control. BOLD response within a frontal network including ACC, rIFG and bilateral insula cortex, was enhanced for 7-token offers compared to 3 and 5-token offers, and thus scaled with the demand for control. The betas for a region in the rIFG (circled in red) are plotted for illustration. Vertical lines represent SEM. * indicates p = < 0.05; n.s. indicates not significant (paired t-tests); see also Inline Supplementary Table S3.

Thus, activity in these regions did not scale with value but instead with the demand for control (Fig. 5). We note that these regions are strongly implicated in cognitive control (Kerns et al., 2004), response inhibition (Aron et al., 2004) and self-regulated choice (Hare et al., 2009).

## Discussion

Both vmPFC and striatum are implicated in computing value for action selection (Brovelli et al., 2011; FitzGerald et al., 2012; Guitart-Masip et al., 2012; Tricomi et al., 2004; Wunderlich et al., 2012), and these regions are differentially activated when individuals choose immediate versus delayed rewards (McClure et al., 2004). Whether this distinction arises from divergent computational roles has remained unclear. Here, we used a computational formalization to address how vmPFC and striatum arbitrate between immediate and long-term values where these are dissociable and can motivate differing actions. Further, by contrasting incorrect and correct decisions we could map the computational mechanisms that contribute towards impulsive or controlled choice respectively.

Previous studies have proposed that choice utilizes a common value system based on vmPFC (Hare et al., 2009), or on a vmPFC–striatal loop (Kable and Glimcher, 2007). Consistent with this, we identified a value representation in vmPFC that takes into account the immediate and delayed consequences of actions. However, in contrast to the common value framework, we identified a separate representation of immediate value in anterior caudate that likely impacts action selection in parallel, and in a fashion that often opposes a course of action endorsed by vmPFC. In this scheme, failures of self-control stem from a degraded representation of long-term value in lateral vmPFC and a concurrent enhancement of immediate value within the anterior caudate. Analogously, successful control is dependent not only on an accurate representation of long-term value in lateral vmPFC, but also an attenuation of immediate value in anterior caudate.

There are several possible explanations for the discrepancy between our finding that the brain represents dual values and previous accounts that it uses a single value system. In the Hare dietary choice paradigm (Hare et al., 2009), subjects chose between a reference food item and alternatives that varied in healthiness and tastiness. The authors then asked whether the BOLD response significantly correlated with taste or health ratings in subjects who demonstrated either high or low capacity for self-control. However, this analysis was confined to the vmPFC, and it is possible that activity in the anterior caudate may have tracked taste ratings in a manner similar to the immediate value representations that we observed in our data. Further, while tastiness and healthiness map onto different outcome modalities, our task considers immediate and long-term value attributes within a single modality.

A second prominent study closely aligned with the single value account utilized an intertemporal choice paradigm to probe preference for rewards at differing time-scales (Kable and Glimcher, 2007). Here, subjects had to choose between an immediately available sum of money and a larger but delayed alternative. Similar to results reported here, Kable and Glimcher found that vmPFC (among other regions) computes the subjective value of the chosen option. However, since the immediate reward was kept constant in their design, it remains unknown whether this value is tracked separately in the brain.

In a follow-up study, the authors modified their paradigm to include trials in which subjects chose between a smaller amount of money paid at a sooner time (but not immediately) and a larger amount paid at a later time (Kable and Glimcher, 2010). Here the authors found that activity within the same value regions as their previous study (Kable and Glimcher, 2007) encodes subjective value on an absolute scale that depends on duration of delay from the present. Further, the authors report a failure to find evidence that activity in these regions is higher when an immediate option is present as compared to when both options are delayed. The latter suggests that a proposed short-sighted neural value system may not simply represent the presence of an immediate reward, but rather may be sensitive to contexts in which the largest available reward is paired with a less favorable outcome in the long-term. Interestingly, the largest offer is always paired with a delay in temporal discounting studies, and in this sense is never immediately available. Another possibility is that the short-sighted system is unable to take into account delayed value components when there is a requirement for these to be inferred. In contrast to this, the delay to reward is explicitly cued in intertemporal choice paradigms. Lastly, the paradigm employed by these authors poses an interesting question regarding whether a proposed short-sighted system is exclusively sensitive to “immediate” reward, or rather to rewards merely closest to the present (though this goes beyond the scope of the present study).

Another important consideration is that unlike the previous studies, our task did not require a choice between two options presented simultaneously. Rather, subjects were required to flexibly approach or avoid an option with both immediate and delayed consequences, spanning both action and valence (Guitart-Masip et al., 2012). This action dependency was adopted so as to more closely resemble natural settings, where self-control often involves arbitration between approach and avoidance, and where the value of choice options often change dynamically. Given that the striatum is heavily implicated in both action and value processing (Guitart-Masip et al., 2014; Rothwell, 2011; Samejima et al., 2005), and that the distinction between these roles is not clearly defined, anterior caudate may in fact integrate value with a propensity to act during go/nogo judgments (Guitart-Masip et al., 2011, 2012; Roesch et al., 2009). In turn, this contribution may be absent in self-control tasks, such as intertemporal choice tasks, that do not pair the prepotent choice (accepting a large immediate reward) with a prepotent action (the execution of a ‘go’ response). Other evidence that task modality can impact value coding comes from a recent finding that switching the frame of reference used for decision-making alters patterns of value coding in the brain (Hunt et al., 2013).

In humans, activity in vmPFC has been shown to include a representation of healthiness in individuals who resist temptation for unhealthy foods (Hare et al., 2009), a finding complimented by evidence that vmPFC acts to integrate multiple components of value (Wunderlich et al., 2012). Further, in rodents, the orbitofrontal cortex has been shown to compute values based on anticipation of latent outcomes (Jones et al., 2012), while patients with bilateral vmPFC lesions demonstrate reduced sensitivity to future consequence and increased reliance on immediate rewards (Bechara et al., 2000). However, to the best of our knowledge, no previous study has demonstrated a value signal in human vmPFC that reflects an overall (long-term) value that is decoupled from short-sighted valuations related to the presence of an immediate reward or a smaller–sooner option. This points to the likelihood that vmPFC draws on contextual information to calculate an overall expectation of value (Hampton et al., 2006; Jones et al., 2012; McDannald et al., 2011; Takahashi et al., 2013), while other valuation regions may only be privy to immediate outcomes.

We found that value coding in a more ventral region of vmPFC is dependent on subjects' baseline ability to appropriately adjust a prepotent response, raising an important question regarding the underlying mechanism. One conjecture is that this region lacks access to representations required for inferring long-term value in impulsive players. This may be related to a weaker functional connectivity between this region of vmPFC and more dorsal prefrontal cortex regions associated with goal-directed control (Hare et al., 2009, 2014). By contrast, value coding in a more lateral region of vmPFC was predictive of upcoming choice in a context requiring self-control. While we can only speculate as to the functional differences between these regions, one possibility is that the ventral portion encodes long-term value regardless of context, whereas the more lateral portion integrates long-term value with additional components that contribute to the action selection process, and is thus more representative of upcoming choice. Interestingly, a recent study has identified a similar pattern of differential reward processing within subregions of vmPFC in non-human primates (Monosov and Hikosaka, 2012).

Our finding that anterior caudate predominantly tracks immediate value is surprising given previous accounts that this region represents the utility of actions by differentiating between positive and negative consequences (Tricomi et al., 2004), or computing values for planned choice (Wunderlich et al., 2012) and future reward prediction (Tanaka et al., 2004). A long-line of animal research has implicated the dorsomedial striatum (the caudate homologue in rodents) in representing the consequences of an animal's actions, with lesions to this region impairing the acquisition of R–O contingencies (Yin et al., 2005). Yet, much of the animal literature relies on devaluation paradigms that utilize immediate outcomes (Balleine and O'Doherty, 2010). Similarly, experiments in humans have implicated anterior caudate in outcome devaluation (Valentin et al., 2007) and in tracking contingencies between actions and outcomes (Tanaka et al., 2008), yet often do not require valuations that integrate immediate and long-term consequences. Thus, one possibility is that both vmPFC and anterior caudate support goals by representing outcomes (Valentin et al., 2007), while vmPFC predominantly receives the input required to calculate long-term value. An alternative interpretation, given a finding that at least some component of the anterior caudate response is explained by long-term value, is that this region contains populations of neurons tuned to either immediate or long-term value respectively.

We note that although we used a model-based tree search to define overall value for the purposes of our analysis, our task cannot differentiate between model-based versus alternate choice strategies. For example, the use of heuristics may be more probable given the complexity of the tree search. Further, subjects' probability of accepting an offer between offer indexes 2 and 3 in a trial (see Fig. 2A, yellow boxes) is somewhat uniform, and this choice pattern is not well-captured by the winning model. Yet our key interest lay in exploring the behavioral and neural consequences of dissociating immediate from overall (long-term) value, and the trade-off model provides corroborative evidence that subjects take both quantities into account. An important follow-up question is whether long-term value is calculated online by projecting into the future, or whether it is cached and retrieved in a model-free framework following a sufficient number of trials.

Our data have a number of implications. Comorbidity between impulsivity and selected psychiatric disorders is well-documented (Moeller et al., 2001), raising an interesting question as to the relationship between the biological substrates of these disorders and the dissociable value representations that we identify. Our task might provide a novel avenue for probing this, including assessing the impact of both behavioral and pharmacological interventions. Finally, given a strong association between affective state and the capacity for self-control, the dual-value framework that we outline could be useful for evaluating the impact of emotion, mood, stress, and other state-dependent factors on the representation of immediate and long-term values, and the resulting impact on decision-making in these contexts.
