# Supplementary material for: Arbitration between controlled and impulsive choices
Source: Neuroimage. 2015 Apr 1;109:206–16. doi: 10.1016/j.neuroimage.2014.12.071 (PMC4349632; doi:10.1016/j.neuroimage.2014.12.071)
Supplement: Inline Supplementary Table S1 [file mmc1.docx]

**Table S1. Best-fitting group-level parameter estimates from the winning model, shown as median and quartiles across subjects.** *τ = inverse temperature, c_1_* ***=*** *value intercept, t_3_* ***=*** *trade-off for value 3, t_5_* ***=*** *trade-off for value 5, t_7_* ***=*** *trade-off for value 7*.

|  | **τ** | **c_1_** | **t_3_** | **t_5_** | **t_7_** |
| --- | --- | --- | --- | --- | --- |
| **25^th^ percentile** | 1.856 | 5.160 | 0.763 | 0.488 | 0.160 |
| **median** | 2.833 | 5.891 | 0.834 | 1.000 | 0.264 |
| **75^th^ percentile** | 3.682 | 6.442 | 0.872 | 1.000 | 0.437 |
