# Supplementary material for: Arbitration between controlled and impulsive choices
Source: Neuroimage. 2015 Apr 1;109:206–16. doi: 10.1016/j.neuroimage.2014.12.071 (PMC4349632; doi:10.1016/j.neuroimage.2014.12.071)
Supplement: Inline Supplementary Table S2 [file mmc2.docx]

**Table S2. Measure of goodness-of-fit, assessed via pseudo r^2^ (**[**Cox and Snell, 1989**](#_ENREF_9)**), for the different models tested (shown as means with 95% confidence intervals).**

| **r^2^** | *immediate* | *optimal* | *1 trade* | *3 trade* |
| --- | --- | --- | --- | --- |
| **95% CI: lower** | -0.144 | 0.162 | 0.389 | **0.416** |
| **mean** | -0.046 | 0.234 | 0.459 | **0.490** |
| **95% CI: upper** | 0.052 | 0.306 | 0.529 | **0.563** |
