# Supplementary material for: Arbitration between controlled and impulsive choices
Source: Neuroimage. 2015 Apr 1;109:206–16. doi: 10.1016/j.neuroimage.2014.12.071 (PMC4349632; doi:10.1016/j.neuroimage.2014.12.071)
Supplement: Inline Supplementary Table S3 [file mmc3.docx]

**Table S3. Regions that track the demand for action control.** Regions where BOLD covaried with the demand for control ({ 0 -1 1 } for offers of token-value 3, 5 and 7 respectively) from *GLM 1*.

| Name of Region | Cluster FWE p value | MNI Coordinates | | | Statistics | |
| --- | --- | --- | --- | --- | --- | --- |
|  |  | x | y | z | t value | Z score |
| Anterior Cingulate | < 0.001 | 10 | 34 | 30 | 5.33 | 4.77 |
| R Supplementary Motor Area |  | 8 | 16 | 66 | 3.69 | 3.42 |
| L Insula | 0.013 | -33 | 15 | 0 | 5.09 | 4.49 |
| R Insula | < 0.001 | 32 | 20 | 2 | 4.91 | 4.36 |
| R Inferior Frontal Gyrus |  | 54 | 12 | 19 | 3.78 | 3.50 |
| R Parietal | < 0.001 | 58 | -43 | 34 | 4.23 | 3.85 |
